# Rebound effects and green growth – An examination of their relationship in a parsimonious equilibrium input-output-framework

**DOI:** 10.1016/j.jclepro.2019.03.296

**Published:** 2019-07-10

**Authors:** Eckehard Rosenbaum

**Affiliations:** European Commission, Joint Research Centre (JRC), Via E. Fermi 2749, Ispra, VA, Italy

## Abstract

Attempts to curb pollution or reduce resource consumption while maintaining growth and thus to achieve what is often referred to as green growth are frequently compromised by rebound effects. That is, the reduction of emissions or resource consumption is less than what would be expected from a technical perspective. Rebound effects may occur because greater efficiency makes the use of a resource cheaper in economic terms and thereby more widespread, or because the demand for green products induces additional demand for brown products. The focus of the present research is to examine from a theoretical-cum-simulation perspective the latter aspect, and the resulting possibility that firms may invest in additional capacity for producing brown rather than green products, even if, initially, there is increasing demand for green products. Such effects could be termed macro-level rebound effects in that there occurrence and significance depends on the properties of the production system as a whole. The purpose of the paper is to develop a parsimonious framework, combining a simple input-output model with some standard macroeconomic relationships between savings, consumption and investment within which macroeconomic rebound effects can be analysed. In doing so, the paper purports to show, undertaking a Monte-Carlo study, that in a broad range of technological configurations, green growth may be feasible in the sense that macroeconomic rebound effects are unlikely to occur. However, depending on the structure of the economy (i.e. depending on the extent to which sectors are green) this result must be qualified: the more sectors exist where green capital is equally productive, the higher is the likelihood of rebound effects. Moreover, it is mainly the productivity of using a good for its own production which determines the likelihood of rebound: The lower the productivity, the higher the likelihood that an increase in the production of that capital good will be eaten up to a large extent by the resulting input requirements of the same industry.

## Highlights

• Rebound effects are examined in a post-Keynesian framework.
• Green capital is assumed to be less productive than brown capital.
• Attempts to foster green growth are not very likely to be undermined by macroeconomic rebound effects.

## 1 Introduction

An increasing number of scientists and policy makers argue that sustainability requires conventional growth to be abandoned (Jackson, 2009) in order to remain within planetary boundaries (Rockström et al., 2009). Concomitantly, growth is perceived to have failed to deliver better lives to everybody as inequalities are rising in many countries (Piketty, 2014) while in a digital world, despite continued economic growth, even more jobs are bound to disappear (Brynjolfsson and McAfee, 2014).

Against this multifaceted background, green growth has become a widely accepted alternative paradigm. Green growth is expected to solve the dilemma of craving for economic growth to fight unemployment and deprivation while acknowledging that unconstrained growth cannot be sustained in a constrained world. Thus “[g]reen growth means fostering economic growth and development while ensuring that natural assets continue to provide the resources and environmental services on which our well-being relies” (OECD, 2011). In the end, Green Growth is so appealing because one may have both – higher production/welfare and a better environment (Rosenbaum, 2017). At the same time, green growth is often assumed to be more labour intensive than conventional growth, thereby helping to curb unemployment by reducing labour shedding through innovation.

However, the popularity of green growth contrasts sharply with a considerable lack of analytical breadth and depth as neither theoretical nor empirical foundations of green growth have been analysed sufficiently (Schmalensee, 2012). In their stead, rather optimistic assumptions regarding the potential for, and the effects of, technological progress are often made. This being said, “[T]he core meaning of the concept of green growth can be simply stated. It is economic growth (growth of gross domestic product or GDP as conventionally understood) which also achieves significant environmental protection.” (Jacobs, 2012). Importantly, the expected levels of environmental protection cannot be achieved with received patterns of growth and this is what makes the concept politically appealing (Jacobs, 2012).

There are a number of causal channels underpinning green growth. Copeland and Taylor (2013) distinguish between scale effects where more production leads to less pollution per unit of production, composition effects where growth leads to cleaner consumption patterns, and technological effects where the need for cleaner technologies is induced by preferences giving higher weight to a clean environment. Related to the latter effect are general efficiency improvements. Such improvements lead to a reduction of the environmental burden because they reduce the demand for a given resource whose use has negative environmental effects. For instance, a more efficient car engine reduces the consumption of fossil fuels per km and therefore any effect that goes along with burning fossil fuels.^1^

**Fig. 1:**
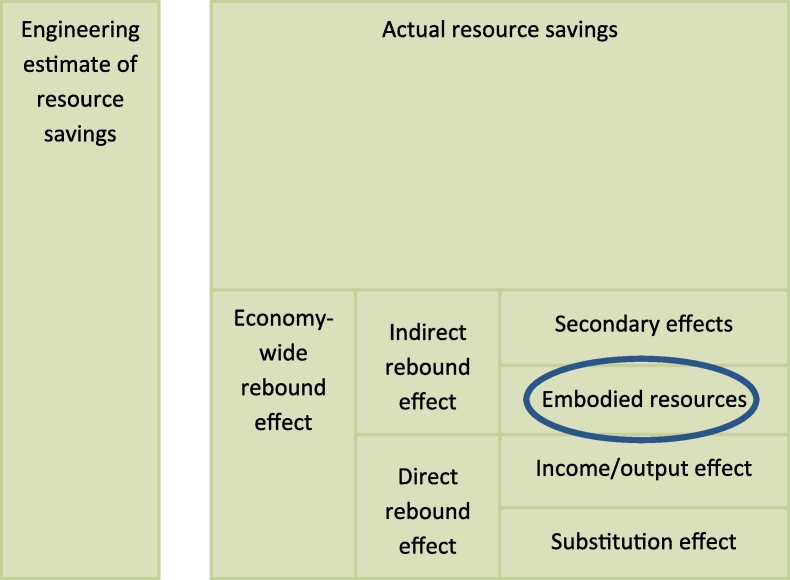
Types of rebound effects (Based on Sorrell (2007)).

Conversely, there are other effects which are prone to undermine green growth. As is well known from the literature, rebound effects often compromise attempts to curb pollution or even reduce resource consumption (Font Vivanco et al., 2016, Hanley et al., 2009, Sorrell, 2010, Wu et al., 2018). Such rebound effects may occur at the micro and the macro level, and they may be either direct or indirect (Jenkins et al., 2011). See Fig. 1 for a systematisation. For instance, rebound effects may take place because greater efficiency makes the use of a resource cheaper in economic terms and thereby more widespread (direct effect). Or because efficiency improvements, which translate into lower prices, lead to higher real income. Such income gains may in turn be spent on the consumption of additional goods and services, thereby reversing at least partially the reduction of pollution or resource consumption (indirect effect). In addition, new household appliances may also generate time savings which lead to higher resource consumption elsewhere (Blum et al., 2018, Jalas, 2009). The size of rebound effects is still a matter of discussion though. For consumers these effects are generally considered to be rather small (Jenkins et al., 2011). By contrast, Saunders (2013) found substantial rebound effects in some, mainly energy intensive, industrial sectors.

In addition to micro-level effects, there are also what could be termed macro-level effects. For instance, if efficiency improvements reduce the demand for a resource, then this may lower the market price of the resource and thereby induce additional demand from consumers or producers which hitherto have not (or less) used that resource (Yang and Li, 2017). Another important macro-level effect is that, in the context of green growth, the production of green products, i.e. products whose use has less harmful effects on the environment, requires potentially harmful inputs from other sectors of the economy. A classic example would be a wind farm which produces electricity and replaces a conventional coal-fired power plant. While the latter produces all sorts of emissions from CO_2_ to sulphur dioxide, which can only be removed using rather expensive technologies, a wind farm is basically emission free. There are other effects on the environment starting from the adverse visual impact of wind farms (Möller, 2006) to the dangers such structures pose for migrating birds (Hüppop et al., 2016), but these are left out of the picture for the moment. The point is nevertheless that from a specific angle, wind farms are greener than coal-fired power plants.

The present research thus starts from the fact that – following the distinction advocated by Kemp-Benedict (2014) – green capital goods, i.e. capital goods with low associated environmental impacts, require for their production all sorts of inputs, many of which may not be “green” at all but “brown”, i.e. with high associated environmental impacts (Helm, 2011). Thus, reverting to the above example, a wind farm may need foundations made of reinforced concrete with steel and cement as major and in fact rather energy and CO_2_ emission intensive inputs. Concomitantly, increased economic activity due to investment in green energy may lead to more demand for energy, not less (Huberty et al., 2011, Sorrell, 2007), due to embodied energy effects (Jenkins et al., 2011). More generally, what appears to be, at a micro level, capital-saving, labour-saving or energy saving, may turn out to be capital-using, labour-using or energy using at the macroeconomic scale (Jenkins et al., 2011). Obviously, such indirect rebound effects do not stop at the level of first-tier suppliers, i.e. producers of steel or cement, but need to take into account also the inputs used by a steel mill or a cement works and ultimately all (direct and indirect) inputs along the whole supply chain (embodied resources). Supply chain effects may therefore not only affect almost all sectors, they may also induce changes of relative prices if the magnitude of demand shifts is sufficient. For these reasons, such indirect effects will also be referred to here as “macroeconomic”. The effects to be explored presently thus differ from studies which use an input-output model to examine emissions from consequential consumption, e.g. Kawajiri et al. (2018).

In the context of a green *growth* agenda, macro-level rebound effects via the supply chain are not only important, however, because “green” capital goods may require “brown” inputs, implying that technological improvements cannot be treated as “manna from heaven” (Barker et al., 2009), but also because additional demand for an input is likely to induce investments if capacity is insufficient or over-utilised in that industry which again require inputs from other sectors of the economy. As a result, the economy may move on a growth path which, despite good intentions, may still be more brown than green. It is the latter possibility which is the focus of the present research.

The papers differs in two respects from, and thus adds to, the existing literature. To begin with and following the tradition established by Jevons (1866), the analysis of rebound effects seems to focus primarily on energy rather than materials. See Freeman (2018). This paper therefore takes a broader approach by analysing a model which distinguishes explicitly between investment and consumption goods. Based on this, growth is not taken as given but insofar as it is driven by investment is endogenous to the approach (Freeman, 2018). Second, neoclassical equilibrium models, e.g. Böhringer and Rivers (2018) and Wei (2007) seek to capture the effects of changing equilibrium prices whereas post-Keynesian equilibrium models seek to capture the effects of changing quantities, the assumption being that prices are rather inflexible because they are determined by the production technology.

Thus the main objective of the paper is to explore whether and under which circumstances a green-growth strategy is likely to be self-supporting in the sense that a higher rate of investment in green capital goods will not be undermined by inducing even higher rates of investment in brown capital goods. In order to facilitate the analysis, however, the paper distinguishes only between brown and green capital goods while remaining agnostic with respect to the properties of the (only) consumption good, i.e. with respect to the issue of green consumption vs. brown investment. This is motivated by the aim to investigate in particular indirect effects in the production chain for which the “greenness” (or not) of the consumption good does not matter since it is by definition not an input.

A major challenge that this line of research has to confront pertains to the amount of data. Including the whole supply chain makes it rather difficult to subject an issue such as green growth to a rigorous theoretical analysis from which general policy conclusion can possibly be drawn. To overcome this problem, the paper develops a parsimonious and arguably consistent equilibrium framework within which the notion of green growth, and in particular the aforementioned supply chain effects, can be analysed comprehensively. For this purpose, three elements are used. The first element is an input-output structure which represents the production technology of the economy and thus embodies the supply-chain effects. The second element consists of a set of basic macroeconomic relationships between savings, consumption and investment in conjunction with an assumption on pricing. These are used to close the model and allow for further aggregation. Last but not least, a Monte-Carlo-Simulation has been used in order to explore the properties of the model for a broad range of parameter values given that an analytical solution could not be found. In the end, the paper purports to show that in a broad range of cases, green growth may be feasible. However, depending on the structure of the economy, i.e. depending on the extent to which sectors are green, the results must be qualified.

The paper is structured as follows. Section II sets out the methodological framework with respect to its 3 major components – capacity utilisation and investments, prices and quantities. Section III presents the results of a numerical investigation of the resulting model while Section IV further examines the results by fitting them to a logit model. Section V discusses the main findings while section VI concludes.

## 2 Methodological framework

In this section, I will first derive the relationship between capacity utilisation and investment in the context of a standard input-output-structure (Part A.). In part B., I will derive prices for the three goods used in the model, again using the input-output-structure. Finally, part C. makes use of some basic macroeconomic relationships in order to close the model.

### 2.1 Capacity utilisation and investment

As pointed out above, whether a product is truly environmentally friendly depends not only on whether the production and use of the final product or services is environmentally friendly, but above all whether the inputs used over the whole supply chain fulfil that criterion. Thus the composition of the capital stock determines to a significant degree the overall environmental impact of economic activity. In the context of a growing economy, the issue is therefore whether investment is predominantly green or brown, all the more so as green investment may *increase* the demand for brown capital services as the above example suggests. If so, this would undermine the move towards a green economy.

In order to address these questions, the paper investigates a simplified economy which consists of three industries. Two of these industries produce capital goods, using capital services (from the same industry and the other industry) and labour as inputs, while the third industry produces a consumption good. By definition, the consumption good is not an input for the other two industries. The two industries which produce capital goods are assumed to differ in two crucial respects^2^:

1. Green capital goods are more environmentally friendly than brown capital goods. More specifically, using green capital goods will be less harmful for the environment than using brown capital goods. No further assumptions will be made, though, about whether environmental friendliness is a one or a multidimensional matter and how much green and brown capital goods differ in this respect as this is not important for the analysis.
2. Following Kemp-Benedict (2014), green capital goods are less productive than brown capital goods. In other words, for producing one unit of output, more green than brown capital services are required. This assumption is important insofar as in its absence, green growth would cease to pose a significant problem in the first place. After all, if green capital was not only less harmful, but also at least equally productive as brown capital, then investors would only invest in green capital and green growth would become self-propagating.

Assuming a Leontief-framework (Leontief, 1970, Miller and Blair, 2009), relative inputs of capital services and labour in each industry are fixed, at least in the short run, and determined by the technology currently in place. Thus within each industry, capital cannot be substituted by labour and one type of capital cannot be substituted by another type of capital. However, this is not meant to imply that firms invest in fixed proportions. Firms in each industry are assumed to invest in the capital good depending on how heavily it is used relative to the available capacity. In other words, investment in either green or brown capital is a function of capacity utilisation (Lavoie, 1992).^3^

(1) $$I_{g,b}=f\left(u\right)$$

This differs from the neoclassical treatment of investment which posits that investment be driven by the marginal productivity of capital. In a Leontief-framework, by contrast, it does not make sense to speak of the marginal productivity of an input since it is impossible to modify its quantity without simultaneously modifying the quantities of all other inputs.

Since neither economies of scale or scope are assumed for each capital good,^4^ capacity is proportional to the capital stock and investment leads to a proportional increase of capacity. Capacity utilisation can therefore be formalised as follows.

(2) $$u_{g}=\frac{\tilde{K_{g}}}{K_{g}}$$

(3) $$u_{b}=\frac{\tilde{K_{b}}}{K_{b}}$$

Here $K_{g}$ and $K_{b}$ are the stocks of green and brown capital measured in physical terms (e.g. the number of hours during which a certain service can be provided during a given reference period) while $\tilde{K_{g}}$ and $\tilde{K_{b}}$ are the demands for green and brown capital services measured in the same unit and for the same period.

The reason for couching the analysis in physical terms is straightforward: the environmental properties of a capital good are intimately related to its physical quantity, much less so and only indirectly to its economic value. Therefore the impact on these quantities determines the overall environmental effect of a green growth strategies.

Effective accumulation of green capital and brown capital (the growth rate of the respective capital stocks due to investment in both types of capital) can then be written as a function of green and brown capital utilization^5^

(4) $$g_{g}\equiv \frac{I_{g}^{e}}{K_{g}}=g\left(u_{g}\right)$$

(5) $$g_{b}\equiv \frac{I_{b}^{e}}{K_{b}}=g\left(u_{b}\right)$$

where $I_{g}^{e}$ and $I_{b}^{e}$ denote *effective* (i.e. deployed instead of planned) investment in green and brown capital. $z_{e}=I_{g}^{e}/I_{b}^{e}$ then denotes the ratio of effective green and brown investment. The reason behind the distinction between planed and deployed investment will become clear shortly. Suffice to note for the moment that the latter also includes the indirect demand effects which result from a given investment plan and must therefore be distinguished from the ratio between planned investment in green and brown capital, which excludes indirect demand effects.

Conceptually, I will distinguish in what follows between the level and the composition of investment. While the *level* of investment and therefore the overall rate of accumulation will be determined following the received albeit very much simplified post-Keynesian approach, i.e. by aggregate demand, the *composition* of investment (the proportion of green and brown capital respectively) is assumed to depend on two factors:

The *primary* factor is *planned* relative investment in green and brown capital:

(6) $$z_{p}\equiv \frac{I_{g}}{I_{b}}$$

$z_{p}$ reflects the structure of investment plans of entrepreneurs and is assumed being driven by factors *exogenous* to the model such as economic policy incentives or more environmentally conscious consumers as a result of which $z_{p}$ increases. A similar effect would occur if there were scale effects in the accumulation of green capital (Kemp-Benedict, 2014) such that green capital becomes more productive (and hence cheaper) if it is added to an already existing sizable stock. However, since this would require rather tedious modifications to the model, the idea is not explored further.

$z_{p}$ must be distinguished from z which describes the actual composition of the capital stock *in situ*.

(7) $$z\equiv \frac{K_{g}}{K_{b}}$$

Obviously, z should change over time if $z_{p}\neq z$. Thus z should increase whenever $z_{p}$ is larger than z and it should decrease whenever $z_{p}$ is smaller than z.^6^

The *secondary* (endogenous) factor, which also occurs later in time, stems from the fact that the structure of demand translates into specific demands for services from the existing capital stock. Given the structure of this capital stock, this implies in turn that different types of capital may be used to different degrees. Thus increased investment in green capital may boost the demand for green capital services, but it will also require additional services from brown capital depending on the production technology in place. Entrepreneurs are assumed not to know the structure of the economy and therefore cannot factor in these indirect effects. The point to be examined is therefore whether the additional demand for capital services is proportional to the exiting capital stock or whether it implies that some capital goods are more intensively used than others.

Production technology can be described by means of a technology matrix A for three products:

(8) $$A=\left[\begin{array}{ccc} a_{11} & a_{12} & a_{13}\\ a_{21} & a_{22} & a_{23}\\ a_{31} & a_{32} & a_{33} \end{array}\right]$$

Element $a_{ij}$ of the matrix describes how much input i is required to produce one unit of output j. Inputs/outputs are green capital (i=1), brown capital (i=2) and a consumption good (i=3). The coefficients are assumed to be constant. Thus there is no technological progress and also no product innovation enabled by more efficient production technologies. These are significant constraints which need to be considered in any medium to long-term analysis (Madlener and Alcott, 2009).

As already point out, it will be assumed that the productivity of green capital is lower than the productivity of brown capital. Within the context of the above technology matrix this means that more green than brown capital services are needed for producing one unit of output. Hence $a_{11}>a_{21}$, $a_{12}>a_{22}$ and $a_{13}>a_{23}$. In addition, no industry can obviously use more of its own output than what it actually produces. Hence $a_{11}<1$ and $a_{22}<1$.

At this point it may be argued that a comparison of physical input-coefficients does not make much sense as it amounts to comparing apples with oranges so to speak. While this argument has to be taken seriously – after all, using a hammer for 1 h would appear to be qualitatively different from using a saw for 1 h, even if the environmental characteristics are comparable –, it does not undermine the focus of the analysis. To see this, note that it is always possible to define a baseline against which the technical coefficients are being compared, if necessary after making some adjustments. Such adjustments would imply that instead of requiring that $a_{11}>a_{21}$, one would require that $\lambda_{1}a_{11}>\lambda_{2}a_{21}$, where $\lambda_{i}$ is an adjustment factor, which seeks to capture qualitative differences and makes physical units comparable. Such an adjustment factor could for instance be a composite indicator of the various non-environmental properties of a capital good.

Assuming that nothing is wasted, an industry i produces what is used for production by the same industry i, by other industries j≠i and what is demanded by both industries in terms of investment goods and households in terms of consumption goods $\left(d_{i}\right)$. Hence production x in industry 1 must be:

(9) $$x_{1}=a_{11}x_{1}+a_{12}x_{2}+a_{13}x_{3}+d_{1}$$

Or

(10) $$\left(1-a_{11}\right)x_{1}-a_{12}x_{2}-a_{13}x_{3}=d_{1}$$

Consequently, production in the remaining two industries must be

(11) $$-a_{21}x_{1}+\left(1-a_{22}\right)x_{2}-a_{23}x_{3}=d_{2}$$

$$-a_{31}x_{1}-a_{32}x_{2}+\left(1-a_{33}\right)x_{3}=d_{3}$$

In matrix notation, these three equations can be written as

(12) $$\left[\begin{array}{ccc} \left(1-a_{11}\right) & -a_{12} & -a_{13}\\ -a_{21} & \left(1-a_{22}\right) & -a_{23}\\ -a_{31} & -a_{32} & \left(1-a_{33}\right) \end{array}\right]\left[\begin{array}{c} x_{1}\\ x_{2}\\ x_{3} \end{array}\right]=\left[\begin{array}{c} d_{1}\\ d_{2}\\ d_{3} \end{array}\right]$$

or

(13) (I−A)x=d

where I is the identity matrix, A is of course the above technology matrix in (8), and x and d are the variables and the final demand vector.

Provided the matrix (I−A) is non-singular, the inputs required for the production of a given final demand vector are then

(14) $$x={\left(I-A\right)}^{-1}d$$

With final demand consisting of three components – investment in green and brown capital and consumption –I have

(15) $$d=\left[\begin{array}{c} I_{g}\\ I_{b}\\ C_{d} \end{array}\right]$$

and

(16) $${\left(I-A\right)}^{-1}=\left[\begin{array}{ccc} b_{11} & b_{12} & b_{13}\\ b_{21} & b_{22} & b_{23}\\ b_{31} & b_{32} & b_{33} \end{array}\right]\equiv B$$

Where for instance

(17) $$b_{11}=\frac{\left(1-a_{22}\right)\left(1-a_{33}\right)-a_{23}a_{32}}{\left(1-a_{11}\right)\left(1-a_{22}\right)\left(1-a_{33}\right)-a_{21}a_{32}a_{13}-a_{31}a_{12}a_{23}-\left(1-a_{11}\right)a_{32}a_{23}-a_{31}\left(1-a_{22}\right)a_{13}-a_{21}a_{12}\left(1-a_{33}\right)}$$

The term in the denominator is the determinant of A (denoted henceforth as |A|) and must be nonzero for there to be a solution.

Capital services and consumption good inputs

(18) $$\left[\begin{array}{c} x_{1}\\ x_{2}\\ x_{3} \end{array}\right]\equiv \left[\begin{array}{c} \tilde{K_{g}}\\ \tilde{K_{b}}\\ C_{p} \end{array}\right]$$

required for the production of given investment and consumption demand are therefore

(19) $$\left[\begin{array}{c} \tilde{K_{g}}\\ \tilde{K_{b}}\\ C_{p} \end{array}\right]=\left[\begin{array}{ccc} b_{11} & b_{12} & b_{13}\\ b_{21} & b_{22} & b_{23}\\ b_{31} & b_{32} & b_{33} \end{array}\right]\left[\begin{array}{c} I_{g}\\ I_{b}\\ C_{d} \end{array}\right]$$

where ${\left[I_{g},I_{b},C_{d}\right]}^{’}$ is the vector of final demand in physical terms. This is discussed in more detail below. Note that the elements of matrix B capture not only the direct effects but also the indirect effects, i.e. the demand for capital services which results from the production of inputs for the production of other inputs, i.e. capital services.

If the consumption good is not an input to the production of the capital goods and of itself (as one would assume given its definition as a consumption good), i.e. $a_{31}=a_{32}=a_{33}=0$, the determinant |A| from equation (17) becomes

(20) $${\left|A\right|}^{\#}=\left(1-a_{11}\right)\left(1-a_{22}\right)-a_{21}a_{12}$$

and therefore

(21) $$b_{33}^{\#}=\frac{\left(1-a_{11}\right)\left(1-a_{22}\right)-a_{12}a_{21}}{\left(1-a_{11}\right)\left(1-a_{22}\right)-a_{21}a_{12}}=\frac{1}{1}=1$$

Moreover, $b_{31}^{\#}$ and $b_{32}^{\#}$ are equal to zero for $a_{31}=a_{32}=a_{33}=0$, implying with (19) and equation (21) that $C_{p}=C_{d}$. In other words, the produced amount of the consumption good always equals the demand for this good since there are no indirect effects. For this reason, the subscripts *p* and *d* can henceforth be dropped.

Keeping in mind the assumption that the consumption good is not an input in its own right, i.e. $a_{31}=a_{32}=a_{33}=0$, I get the remaining coefficients $b_{ij}$ of matrix B (equations (22), (23), (24), (25), (26), (27) in the annex).

Using (19) gives us the effective demand in physical terms for green and brown capital services taking into account the indirect effects in the economy:

(28) $$\tilde{K_{g}}=b_{11}\cdot I_{g}+b_{12}\cdot I_{b}+b_{13}\cdot C$$

(29) $${\tilde{K}}_{b}=b_{21}\cdot I_{g}+b_{22}\cdot I_{b}+b_{23}\cdot C$$

By dividing (28) and (29) by $K_{g}$ and $K_{b}$ respectively, I obtain the rates of green and brown capital utilization for a given vector of planned demand.

(30) $$u_{g}=\frac{b_{11}\cdot I_{g}+b_{12}\cdot I_{b}+b_{13}\cdot C}{K_{g}}$$

(31) $$u_{b}=\frac{b_{21}\cdot I_{g}+b_{22}\cdot I_{b}+b_{23}\cdot C}{K_{b}}$$

If the accumulation rates of green and brown capital are as argued above functions of capacity utilisation (equations (4), (5)), then so is $z_{e}$. That is, relative investment in green and brown capital depends on capacity utilisation for each type of capital.

To see this more clearly, note that equations (4), (5) can also be written as

(32) $$I_{g}^{e}=K_{g}g\left(u_{g}\right)$$

and

(33) $$I_{b}^{e}=K_{b}g\left(u_{b}\right)$$

Dividing equation (32) by equation (33) gives, with $z=K_{g}/K_{b}$ as the relative capital stock or capital ratio,

(34) $$\frac{I_{g}^{e}}{I_{b}^{e}}=\frac{K_{g}g\left(u_{g}\right)}{K_{b}g\left(u_{b}\right)}=z\frac{g\left(u_{g}\right)}{g\left(u_{b}\right)}=z_{e}$$

or (replacing $\frac{g\left(u_{g}\right)}{g\left(u_{b}\right)}$ by $f\left(\frac{u_{g}}{u_{b}}\right)$)

(35) $$z_{e}=zf\left(\frac{u_{g}}{u_{b}}\right)$$

The interpretation of this equation is as follows: $z_{e}$ (the induced ratio of green to brown investment) differs from z (the actual capital ratio) if f(.)≠1. As a consequence, however, z will change over time since the composition of investment differs now from the composition of the original capital stock. For the sake of simplicity, I will assume that this is the case whenever

(36) $$\frac{u_{g}}{u_{b}}\neq 1$$

i.e. whenever relative capacity utilisation differs from 1.^7^ In other words, whenever capacity utilisation differs between the green and brown sector, entrepreneurs are assumed to change their investment plans. The direction of change then depends on the absolute value of $u_{g}/u_{b}$.

Using equations (30), (31) for capacity utilisation, relative capacity utilisation $u_{g}/u_{b}$ differs from 1 whenever

(37) $$\frac{u_{g}}{u_{b}}=\frac{\frac{b_{11}\cdot I_{g}+b_{12}\cdot I_{b}+b_{13}\cdot C}{K_{g}}}{\frac{b_{21}\cdot I_{g}+b_{22}\cdot I_{b}+b_{23}\cdot C}{K_{b}}}=\frac{b_{11}\cdot I_{g}+b_{12}\cdot I_{b}+b_{13}\cdot C}{b_{21}\cdot I_{g}+b_{22}\cdot I_{b}+b_{23}\cdot C}\cdot \frac{1}{z}\neq 1$$

i.e., whenever the ratio of capital services required by producing investment and consumption good(s) differs from the composition of the capital stock.

### 2.2 Prices

The analysis undertaken so far has used physical values for quantifying capital, investment and consumption. To continue, I need monetary values for $I_{g},\;I_{b}$ and *C*_*d*_ in order to both simplify the relationships between demand components and for aggregating these components further. To get monetary values, I obviously need prices and also need to make further assumption about quantities. In order to obtain prices, I will apply the standard mark-up pricing hypothesis to the model whereas in order to obtain quantities, I will use some basic macroeconomic relationships between savings, consumption and investment.

I begin with prices. In the context of the above input-output-structure and assuming mark-up pricing, the price vector $p=\left[p_{g},p_{b},p_{c}\right]$ for the two capital goods and the consumption good is determined by cost of production encompassing capital services and labour (see Miller and Blair (2009)) plus a mark-up m on these costs. The mark-up reflects the degree of competition on the respective market and is a widespread assumption in post-Keynesian economics (Lavoie, 1992, Shapiro and Sawyer, 2003). With *w* as the exogenously determined wage rate and **l** as a vector of labour inputs $l^{’}=\left[l_{g},l_{b},l_{c}\right]$ I obtain:

(38) p=(pA+wl)(1+m)

Hence

(39) $$p=wl{\left({\left(1+m\right)}^{-1}I-A\right)}^{-1}$$

With, for ease of notation, $B^{*}\equiv {\left({\left(1+m\right)}^{-1}I-A\right)}^{-1}$ I get

(40) $$p=wlB^{*}$$

Using this I can obtain the following expressions for the three prices in the model:

(41) $$p_{g}=w\left(l_{g}b_{11}^{*}+l_{b}b_{21}^{*}\right)$$

(42) $$p_{b}=w\left(l_{g}b_{12}^{*}+l_{b}b_{22}^{*}\right)$$

(43) $$p_{c}=w\left(l_{g}b_{13}^{\text{*}}+l_{b}b_{23}^{\text{*}}+l_{c}\right)$$

As can be seen, the prices depend only on the wage rate, the labour input coefficients and the composite parameter $B^{*}$ , which *inter alia* includes the mark-up. Before discussing quantities, it should be noted that, in the current framework, prices are not given because the marginal impact of each consumer or producer is small, but because prices are determined by cost of production. Thus the level of demand relative to supply has no impact. This distinguishes the current analysis from analyses which are placed in the context of the textbook demand and supply model and merely assume households to be price takers, e.g. Thomas and Azevedo (2013a) and Thomas and Azevedo (2013b). Arguably, cost of production provide a better explanation of prices on markets where produced goods rather than stocks are traded and where in equilibrium prices must cover costs (Lavoie, 1992).

### 2.3 Quantities

The purpose of this section is to derive a relationship between investment and consumption, which allows us to simplify the model further. Assuming a uniform savings rate s while requiring that (the value of) investment equals savings, i.e. s⋅p⋅Y=p⋅I and therefore $=\frac{p\cdot I}{s}$ , the value of consumption $p_{c}\cdot C$ can be expressed as a fraction of the total value of investment^8^

(44) $$p\cdot Y\left(1-s\right)=p_{C}\cdot C$$

$$\frac{1-s}{s}p\cdot I=p_{C}\cdot C$$

where p is the GDP deflator. From the definition of $z_{p}$ it follows that

(45) $$I_{b}=\frac{I_{g}}{z_{p}}$$

And therefore

(46) $$p\cdot I=p_{g}\cdot I_{g}+p_{b}\cdot I_{b}=p_{g}\cdot I_{g}+p_{b}\cdot \frac{I_{g}}{z_{p}}=I_{g}\left(p_{g}+\frac{p_{b}}{z_{p}}\right)$$

Using (41) to (43) together with (44) to (46), equation (37) becomes

(47) $$\frac{u_{g}}{u_{b}}=\frac{b_{11}+\frac{b_{12}}{z_{p}}+b_{13}\frac{1-s}{s}\frac{l_{g}b_{11}^{*}+l_{b}b_{21}^{*}+\frac{l_{g}b_{12}^{*}+l_{b}b_{22}^{*}}{z_{p}}}{l_{g}b_{13}^{*}+l_{b}b_{23}^{*}+l_{c}}}{b_{21}+\frac{b_{22}}{z_{p}}+b_{23}\frac{1-s}{s}\frac{l_{g}b_{11}^{*}+l_{b}b_{21}^{*}+\frac{l_{g}b_{12}^{*}+l_{b}b_{22}^{*}}{z_{p}}}{l_{g}b_{13}^{*}+l_{b}b_{23}^{*}+l_{c}}}\frac{1}{z}$$

Inserting (47) into (35) gives

(48) $$z_{e}=zf\left(\frac{b_{11}+\frac{b_{12}}{z_{p}}+b_{13}\frac{1-s}{s}\frac{l_{g}b_{11}^{*}+l_{b}b_{21}^{*}+\frac{l_{g}b_{12}^{*}+l_{b}b_{22}^{*}}{z_{p}}}{l_{g}b_{13}^{*}+l_{b}b_{23}^{*}+l_{c}}}{b_{21}+\frac{b_{22}}{z_{p}}+b_{23}\frac{1-s}{s}\frac{l_{g}b_{11}^{*}+l_{b}b_{21}^{*}+\frac{l_{g}b_{12}^{*}+l_{b}b_{22}^{*}}{z_{p}}}{l_{g}b_{13}^{*}+l_{b}b_{23}^{*}+l_{c}}}\frac{1}{z}\right)$$

Hence relative induced investment depends on the parameters of the technology matrix, the savings’ rate, labour inputs and via prices on the mark-up. It does not, however, depend on the absolute level of investment in any specific capital good.

As argued above, (i) $f^{’}>0$ and (ii) f(1)=1. That is, induced investment will deviate from the composition of the capital stock if the relative demand for capital services multiplied by the inverse of z (i.e. relative capacity utilisation) is smaller or larger than one. (i) is fulfilled as long as f is a function that increases steadily in the term in brackets. For (ii) to be the case, a functional form that ensures (ii) is

(49) $$f=\frac{b_{11}+\frac{b_{12}}{z_{p}}+b_{13}\frac{1-s}{s}\frac{l_{g}b_{11}^{*}+l_{b}b_{21}^{*}+\frac{l_{g}b_{12}^{*}+l_{b}b_{22}^{*}}{z_{p}}}{l_{g}b_{13}^{*}+l_{b}b_{23}^{*}+l_{c}}}{b_{21}+\frac{b_{22}}{z_{p}}+b_{23}\frac{1-s}{s}\frac{l_{g}b_{11}^{*}+l_{b}b_{21}^{*}+\frac{l_{g}b_{12}^{*}+l_{b}b_{22}^{*}}{z_{p}}}{l_{g}b_{13}^{*}+l_{b}b_{23}^{*}+l_{c}}}\frac{1}{z}$$

Using this assumption, (48) then becomes

(50) $$z_{e}=\frac{b_{11}+\frac{b_{12}}{z_{p}}+b_{13}\frac{1-s}{s}\frac{l_{g}b_{11}^{*}+l_{b}b_{21}^{*}+\frac{l_{g}b_{12}^{*}+l_{b}b_{22}^{*}}{z_{p}}}{l_{g}b_{13}^{*}+l_{b}b_{23}^{*}+l_{c}}}{b_{21}+\frac{b_{22}}{z_{p}}+b_{23}\frac{1-s}{s}\frac{l_{g}b_{11}^{*}+l_{b}b_{21}^{*}+\frac{l_{g}b_{12}^{*}+l_{b}b_{22}^{*}}{z_{p}}}{l_{g}b_{13}^{*}+l_{b}b_{23}^{*}+l_{c}}}$$

Thus induced investment can be expressed as a function of the parameters of the technology matrix and labour inputs, the savings rate and the planned share of green capital investment expressed in real terms. Note that whether the composition of induced investment differs from the composition of the initial capital stock is independent of the actual value of z. The reason is that z enters the equation for capacity utilisation and is the basis to which new capital is added.

Importantly, z will not only change over time if $z_{p}\neq z$, i.e. if the composition of planned investment differs from the initial composition of the capital stock z, but also if $z_{e}\neq z_{p}$. That is, if induced investment differs from planned investment. Moreover, if $z_{e}\neq z_{p}=z$, i.e. if the composition of induced investment $z_{e}$ differs from the composition of neutral investment $z_{p}=z$, then the composition of the capital stock also changes even if planned investment reflects the composition of the capital stock. This effect, if it occurs, is a good example for the difference between economic effects at micro and macro scale: whatever happens as a *direct* consequence of the behaviour of economic actors may have quite different and hence unexpected *indirect* consequences.

To better see the indirect effects of investment, I will assume that $z_{p}=z$ and then try to find a value for z, henceforth referred to as $z_{n}$, for which the term in bracket equals 1, i.e. a composition of the capital stock, which is such that, if investment reflects that composition accurately, the composition will be reproduced. Doing so results in a quadratic equation (see Annex). The positive solution of this equation is the value of z and $z_{p}$, which ensures simultaneously that, even taking into account indirect effects, the composition of the capital stock does not change.

In the following section, z will be examined numerically together with the effects of various parameter constellations on $z_{e}$. The aim is to see how much $z_{p}$ and $z_{n}$ differ on average.

## 3 Numerical simulations

In this section, the effects of various parameter constellations on $z_{e}$ will be investigated. To begin with, what is the effect of changes in the initially planned composition of investment $z_{p}$ on $z_{e}$ ? Closer inspection of equation (50) shows that the effect is indeed ambiguous. It depends on the values of the parameters in the technology matrix $a_{ij}$ and the resulting composite parameters $b_{ij}$. However, no clear-cut analytical result could be derived.

The ultimate effects of changes in specific parameters of the technology matrix are equally ambiguous. While the derivatives of the composite parameters $b_{ij}$ with respect to $a_{ij}$ have always an unequivocal sign, the effects on $u_{g}/u_{b}$ depends on the relative size of the derivatives. Again no unambiguous result could be found.

To investigate matters further, a Monte-Carlo study has been undertaken. A Monte-Carlo study is particularly useful where there is considerable uncertainty about the numerical values of key variables and parameters so that an analytical solution based on any pre-chosen value does not produce reliable results. In the present case, therefore, the parameters of the technology matrix have been randomized considering that these are the most important source of uncertainty and so has $z_{p}$ taking into account the aforementioned constraints but without always imposing that green capital is less efficient than brown capital. By contrast, the savings rate s has been kept constant and is assumed to be 0.25 while the mark-up m has been set at 0.1.^9^

The technological coefficients have been drawn at random from a uniform distribution between 0 and 1 with mean 0.5 whereas $z_{p}$ has been drawn from a lognormal distribution with mean 1 and a standard deviation of 1.3.^10^ The first assumption implies that all technologies are equally likely whereas the second assumption means that both very high and very low shares of green or brown capital respectively are considered to be unlikely. Note again that the results are independent of the initial value of z.

The study, which involved 100000 runs,^11^ has led to the following results depending on the number of sectors in which green capital is less productive (Table 1)^12^:

**Table 1:** Simulation results.

Data Analysis (mark-up *m* = 0.1, *z*_*p*_ chosen at random, 100000 cases)					
Total number of cases with positive prices	Number of cases with positive prices and green capital being less productive				
	in all sectors	in 2 sectors	in 1 sector	in none of the sectors	
37374	3756	13147	14823	5648	
	**Of which induced investment is greener than planned investment**				**Total**
	3379	10344	9099	2347	25169
Percentages					
37%	90%	79%	61%	42%	67%
	**Number of cases with *z***_***p***_** >* z***_***n***_**, positive prices and green capital being less productive**				**Total**
	**in 3 sectors**	**in 2 sectors**	**in 1 sector**	**in none of the sectors**	
	0	12	102	184	298
Percentages					
	0.0%	0.1%	0.7%	3.3%	0.8%

Green capital is less productive in all sectors

1. Induced investment is mostly (in 90% of cases) greener than planned investment. However, in 10% of cases, induced investment is browner than planned investment. Thus the move to a greener economy is in these cases and in that sense self-defeating.
2. The likelihood of induced investment being browner than planned investment increases as the planned share of green relative to brown investment ($z_{p}$) increases: up until a value of $z_{p}$ ≈1.2, induced investment is always greener than planned investment. As the share of green investment increases further, the likelihood of induced investment being greener decreases and for relatively large values of $z_{p}$ it is basically nill.
3. For the parameters investigated here (green economy), the neutral investment share $z_{n}$ is larger than any (randomly drawn) planned investment $z_{p}$, implying that only a relatively high share of green investment will lead to a reproduction of the composition of the capital stock.

Green capital is less productive in most sectors

1. Induced investment is predominantly (in 79% of cases) greener than planned investment. However, in 21% of cases, induced investment is browner than planned investment. Thus the move to a greener economy is in these cases (and in that sense) partly self-defeating.
2. The likelihood of induced investment being browner than planned investment increases again with the planned share of green relative to brown investment ($z_{p}$). However, unlike in the case of a fully green economy, the bound below which induced investment is always greener is much smaller ( $z_{p}$ ≈0.2).^13^
3. For the parameters investigated here (i.e. a mostly green economy), neutral investment $z_{n}$ is mostly larger than planned investment $z_{p}$, implying that only a relatively high share of green investment will lead to a reproduction of the composition of the capital stock.

Green capital is mostly as productive as brown capital

1. Induced investment is in a majority of cases (61%) greener than planned investment. However, in 39% of cases, induced investment is browner than planned investment. Thus the move to a greener economy is in these cases (and in that sense) self-defeating.
2. The likelihood of induced investment being browner than planned investment increases again with the share of green relative to brown investment ($z_{p}$). However, unlike in the case of a fully green economy, no significant lower bound can be established below which induced investment is always greener.
3. For the parameters investigated here (mostly brown economy), neutral investment $z_{n}$ is mostly larger than planned investment $z_{p}$, implying that only a relatively high share of green investment will lead to a reproduction of the composition of the capital stock.

Green capital is at least as productive as brown capital

1. Now, induced investment is only in a minority of cases (42%) greener than planned investment. In a majority of cases, induced investment is less green than planned investment. Put differently, if green capital is at least as productive as brown capital, then induced investment does not support the move towards a green economy. It has to be noted though that due to its equivalent productivity across the economy, green investment does not need to be subsidized either.
2. The likelihood of induced investment being browner than planned investment continues to increase with the share of planned green relative to brown investment.
3. For the parameters investigated here (brown economy), neutral investment $z_{n}$ is mostly larger than planned investment $z_{p}$, implying that only a relatively high share of green investment will lead to a reproduction of the composition of the capital stock.

## 4 Estimating a logit model

In order to gain some insights about the role of specific parameters of the above model, the simulation data have been fitted to a simple logit model with the probability of $z_{e}>z_{p}$, that is, of induced investment being “greener” than planned investment, as the endogenous variable. The results of this exercise are reported in Table 2.

**Table 2:** Results of the logit regression.

	coeff b	s.e.	Wald	p-value
Intercept	7.59	0.27	798.66	1.0531E-175
*a*_*11*_	9.31	0.30	942.85	4.76E-207
*a*_*12*_	4.60	0.21	503.02	2.0926E-111
*a*_*13*_	2.49	0.17	210.10	1.30572E-47
*a*_*21*_	−6.52	0.23	776.43	7.1837E-171
*a*_*22*_	−9.43	0.29	1051.56	1.1161E-230
*a*_*23*_	−3.12	0.18	309.09	3.44687E-69
$z_{p}$	−5.45	0.12	1935.45	0

Accordingly, the largest impact in absolute terms, although with opposite signs, stems from the two parameters $\left(a_{11},a_{22}\right)$ which capture the impact of the use of a capital good in its own production, i.e. how much of the services which this capital good provides is necessary for its own production. This should not come as a surprise insofar as the quantity of a capital service that is used in the production of the same capital good determine how much is left for production in other industries. Thus, the more green capital services are used in the production of green capital goods, the less productive is green capital.

Conversely, the impact for the brown capital good is large and negative because the more brown capital services are used in the production of brown capital goods, the less productive is brown capital and the more productive in relative terms becomes green capital. The same is true for the parameters which capture the productivity of green and brown capital in the production of the other capital good $\left(a_{12},a_{21}\right)$ , although the effect is now smaller in absolute terms.

The smallest effect can be observed for the consumption good. This is due to the fact that there are no indirect effects from the consumption good on the production of capital goods given that the consumption good is not an input in itself.

## 5 Discussion

The results reported above underpin to a large extent a green-growth strategy. Efforts to foster green growth in an economy where green capital is mostly less productive than brown capital are not likely to be undermined by macroeconomic rebound effects, i.e. by leading to relatively more investment in brown capital goods. The simulation study has found such effects in only 10% of all cases and allowing technologies to differ substantially. This figure increases to approximately 20% if green capital is more productive than brown capital in one sector. Both cases together then account for 81% of all cases. All in all, this appears to be encouraging for proponents of green growth, all the more so as the results holds for a broad spectrum of investment portfolios.

Nevertheless, some rebound effects may occur, and this is in line with findings such as those reported by Freire-González and Font Vivanco (2017), which suggest that improvements in energy efficiency may trigger relevant changes in the consumption of other resources. These changes may be both positive (implying co-benefits) or negative (implying backfire) depending on the structure of consumption and the input-output-structure of each industry. Thus to unconditionally pursue efficiency improvements as an end it itself without taking into account adverse effects appears to be problematic (Schaefer and Wickert, 2015). The present analysis should draw the attention of policy makers to this possibility. It should make them aware of the potential risks of fostering efficiency improvements in the context of an interrelated production structure where many products are both outputs and inputs.

Using the simulation results as inputs for the estimation of a logit regression leads to another noteworthy result. Accordingly, it is predominantly the productivity of a capital good in its own production which determines how productive that capital good is relative to other capital goods and whether, as a result, rebound effects occur. The reason for this finding can be sketched as follows: if a capital good requires a lot of its own input for its own production, then an increase in the production of that capital good will be eaten up to a large extent by the resulting input requirements of the same sector. By contrast, if a capital good requires for its production only a small amount of the services provided by the very same good, then comparatively more is left for use in other sector should the production of this capital good be expanded.

The above results change dramatically once the assumption of lower productivity of green capital goods is dropped altogether, be it because of further technological progress or economies of scale. If brown capital goods are assumed to be less productive in every sector, then rebound effects are *more* likely to occur in the context of a green growth strategy. The reason is that with lower productivity of brown capital goods, any increase of green investment will trigger a proportionally higher demand for brown capital services as inputs for the production of green capital. At the same time, there is no reason anymore why green investment needs economic policy support.

This finding also highlights one of the problems of using input output tables. I/O tables which are no longer up to date, for instance because undertaking surveys is resource intensive, cannot produce reliable results. This is why methods for projecting I/O tables have gained increasing support in the literature (Valderas-Jaramillo et al., 2018). However, in a technologically dynamic environment, I/O coefficients are subject to continuous changes. To the extent that technological progress is difficult to predict in the longer run, simulation results based on constant coefficient have to be interpreted with caution as technological changes may lead to changes in relative efficiency.

In addition, it is of obvious importance what kind production technology is assumed. In a neoclassical setting, for instance, investment would be driven by the marginal productivity of an input. And if the assumed lower average productivity of green capital would translate into a lower *marginal* productivity, then that would hinder green investment rather than pushing it. So the results are likely to be different from those obtained here.

## 6 Conclusions

Given the heavy reliance in many countries on efficiency improvements as a key driver of greening the economy, understanding rebound effects is clearly important (Thomas and Azevedo, 2013a), all the more so as policy makers have been rather reluctant to address rebound effects (Font Vivanco et al., 2016).

This paper has examined the problem of macro-economic rebound effects in the context of an input-output model in combination with key features of a post-Keynesian growth model. Its main objective was to explore whether and under which circumstances a green-growth strategy is likely to be self-supporting in the sense that a higher rate of investment in green capital goods will not be undermined by inducing even higher rates of investment in brown capital goods. In so doing, the paper focuses on one of the features determining the turn-over of the capital stock and hence an issue that has not received a lot of attention in the debate on rebound effects (Madlener and Alcott, 2009).

The research underpins to a large extent a green-growth strategy. Attempts by policy makers to foster green growth in an economy where green capital is mostly less productive than brown capital are not likely to be undermined by macroeconomic rebound effects, i.e. by leading to relatively more investment in brown capital goods. The estimation of a logit regression leads to another interesting result. Accordingly, it is predominantly the productivity of a capital good in its own production which determines how productive that capital good is relative to other capital goods and whether, as a result, rebound effects occur.

Obviously, these are very general results and have to be interpreted with caution due to the very simplified structure of our economy both in terms of the number of industries and the assumed production technology. Nevertheless, for the technology assumed here, the broad results seem to hold for a wide range of parameter values as evidenced by the regression results. The results are in this sense arguably rather robust and add noteworthy nuances to the existing literature on rebound effects.

Future research should extend the current analysis by using more fine-grained and empirically grounded data on the input-output structure of the economy and by implementing a more sophisticated macroeconomic model. The former would further reduce uncertainty while a more elaborate macro model would add to the realism of the analysis.

## Figures and Tables

**Table 7-1:** Notations

Notation	Description	Comments
$I_{g,b}$	Investment in green or brown capital	Endogenous variable
$u_{i}$	Capacity utilisation	Endogenous variable
$K_{g}$	Stock of green capital	Exogenous variable, no further assumptions
$K_{b}$	Stock of brown capital	Exogenous variable, no further assumptions
$\tilde{K_{g}}$	Demand for green capital services	Endogenous variable
$\tilde{K_{b}}$	Demand for brown capital services	Endogenous variable
$g_{g}$	Accumulation rate for green capital	Endogenous variable
$g_{b}$	Accumulation rate for brown capital	Endogenous variable
$z_{p}$	Planned ratio of green to brown investment	Exogenous variable following a lognormal distribution
z	Actual composition of the capital stock	Exogenous variable, no further assumptions
$a_{ij}$	Input i required to produce one unit of output j	Exogenous variable, values between 0 and 1
$x_{i}$	Production in industry i	Endogenous variable
$d_{i}$	Demand for product i	Exogenous variable, no further assumptions
$C_{p}$	Produced amount of consumption good	Endogenous variable
$C_{d}$	Demand for consumption good	Endogenous variable
$b_{ij}$	Total input i required to produce one unit of output j	Exogenous variable
$z_{e}$	Induced ratio of green to brown investment	Endogenous variable
$p_{i}$	Price of good i	Endogenous variable
$l_{i}$	Labour input for the production of good i	Exogenous variable, no further assumptions
m	Mark-up	Exogenous variable, assumed value 0.1
w	Wage rate	Exogenous variable, no further assumptions
s	Savings rate	Exogenous variable, no further assumptions
p	GDP deflator	Exogenous variable, no further assumptions

**Table 7-2:** Explanation of key terms

Term	Explanation
Capital good	A type of good that provides services for the production of other goods
Consumption good	A type of good that does not provide services for the production of other goods but is only used for consumption
Stock of green capital	The aggregate of green capital goods
Stock of brown capital	The aggregate of brown capital goods
Green capital good	A capital good with low associated environmental impacts
Brown capital good	A capital good with high associated environmental impacts
Green investment	Any addition to the stock of green capital
Brown investment	Any addition to the stock of brown capital
Mark-up	Relative difference between costs and selling price

## Annex 7.

### 7.2. Coefficients of matrix B

(22) $$b_{11}^{\#}=\frac{1-a_{22}}{\left(1-a_{11}\right)\left(1-a_{22}\right)-a_{21}a_{12}}$$

(23) $$b_{12}^{\#}=\frac{a_{12}}{\left(1-a_{11}\right)\left(1-a_{22}\right)-a_{21}a_{12}}$$

(24) $$b_{13}^{\#}=\frac{a_{12}a_{23}+a_{13}\left(1-a_{22}\right)}{\left(1-a_{11}\right)\left(1-a_{22}\right)-a_{21}a_{12}}$$

(25) $$b_{21}^{\#}=\frac{a_{21}}{\left(1-a_{11}\right)\left(1-a_{22}\right)-a_{21}a_{12}}$$

(26) $$b_{22}^{\#}=\frac{1-a_{11}}{\left(1-a_{11}\right)\left(1-a_{22}\right)-a_{21}a_{12}}$$

(27) $$b_{23}^{\#}=\frac{a_{13}a_{21}+a_{23}\left(1-a_{11}\right)}{\left(1-a_{11}\right)\left(1-a_{22}\right)-a_{21}a_{12}}$$

### 7.3. Derivation of $z_{n}$

$z_{n}$ is the positive solution of equation (51) as can be seen from (52).

(51) $$z_{1,2}=\frac{-\beta \pm \sqrt{\beta^{2}-4\alpha \gamma }}{2\alpha }$$

(52)

$$zb_{11}+b_{12}+b_{13}\frac{1-s}{s}\frac{zl_{g}b_{11}^{*}+zl_{b}b_{21}^{*}+l_{g}b_{12}^{*}+l_{b}b_{22}^{*}}{l_{g}b_{13}^{*}+l_{b}b_{23}^{*}+l_{c}}=z^{2}b_{21}+zb_{22}+zb_{23}\frac{1-s}{s}\frac{zl_{g}b_{11}^{*}+zl_{b}b_{21}^{*}+l_{g}b_{12}^{*}+l_{b}b_{22}^{*}}{l_{g}b_{13}^{*}+l_{b}b_{23}^{*}+l_{c}}$$

$$zb_{11}+b_{12}+zb_{13}\frac{1-s}{s}\frac{l_{g}b_{11}^{*}+z_{b}b_{21}^{*}}{l_{g}b_{13}^{*}+l_{b}b_{23}^{*}+l_{c}}+b_{13}\frac{1-s}{s}\frac{l_{g}b_{12}^{*}+l_{b}b_{22}^{*}}{l_{g}b_{13}^{*}+l_{b}b_{23}^{*}+l_{c}}=z^{2}\left(b_{21}+b_{23}\frac{1-s}{s}\frac{l_{g}b_{11}^{*}+l_{b}b_{21}^{*}}{l_{g}b_{13}^{*}+l_{b}b_{23}^{*}+l_{c}}\right)+zb_{22}+zb_{23}\frac{1-s}{s}\frac{l_{g}b_{12}^{*}+l_{b}b_{22}^{*}}{l_{g}b_{13}^{*}+l_{b}b_{23}^{*}+l_{c}}$$

$$0=z^{2}\left(b_{21}+b_{23}\frac{1-s}{s}\frac{l_{g}b_{11}^{*}+l_{b}b_{21}^{*}}{l_{g}b_{13}^{*}+l_{b}b_{23}^{*}+l_{c}}\right)-zb_{13}\frac{1-s}{s}\frac{l_{g}b_{11}^{*}+z_{b}b_{21}^{*}}{l_{g}b_{13}^{*}+l_{b}b_{23}^{*}+l_{c}}+zb_{22}-zb_{11}+zb_{23}\frac{1-s}{s}\frac{l_{g}b_{12}^{*}+l_{b}b_{22}^{*}}{l_{g}b_{13}^{*}+l_{b}b_{23}^{*}+l_{c}}-b_{13}\frac{1-s}{s}\frac{l_{g}b_{12}^{*}+l_{b}b_{22}^{*}}{l_{g}b_{13}^{*}+l_{b}b_{23}^{*}+l_{c}}-b_{12}$$

$$=z^{2}{\left(b_{21}+b_{23}\frac{1-s}{s}\frac{l_{g}b_{11}^{*}+l_{b}b_{21}^{*}}{l_{g}b_{13}^{*}+l_{b}b_{23}^{*}+l_{c}}\right)}_{21}+z\left(b_{22}+b_{23}\frac{1-s}{s}\frac{l_{g}b_{12}^{*}+l_{b}b_{22}^{*}}{l_{g}b_{13}^{*}+l_{b}b_{23}^{*}+l_{c}}-b_{13}\frac{1-s}{s}\frac{l_{g}b_{11}^{*}+z_{b}b_{21}^{*}}{l_{g}b_{13}^{*}+l_{b}b_{23}^{*}+l_{c}}-b_{11}\right)-\left(b_{13}\frac{1-s}{s}\frac{l_{g}b_{12}^{*}+l_{b}b_{22}^{*}}{l_{g}b_{13}^{*}+l_{b}b_{23}^{*}+l_{c}}+b_{12}\right)$$

$$z^{2}\underset{\alpha }{\left(b_{21}+b_{23}\frac{1-s}{s}\frac{l_{g}b_{11}^{*}+l_{b}b_{21}^{*}}{l_{g}b_{13}^{*}+l_{b}b_{23}^{*}+l_{c}}\right)}+z\underset{\beta }{\left(b_{22}+b_{23}\frac{1-s}{s}\frac{l_{g}b_{12}^{*}+l_{b}b_{22}^{*}}{l_{g}b_{13}^{*}+l_{b}b_{23}^{*}+l_{c}}-b_{13}\frac{1-s}{s}\frac{l_{g}b_{11}^{*}+z_{b}b_{21}^{*}}{l_{g}b_{13}^{*}+l_{b}b_{23}^{*}+l_{c}}-b_{11}\right)}-\underset{\gamma }{\left(b_{13}\frac{1-s}{s}\frac{l_{g}b_{12}^{*}+l_{b}b_{22}^{*}}{l_{g}b_{13}^{*}+l_{b}b_{23}^{*}+l_{c}}+b_{12}\right)}=0$$

## Footnotes

1 The introduction of catalytic converters, by contrast, has improved the eco-efficiency of cars in some respects but not their overall efficiency.

2 The approach developed here thus differs from, but also complements, a perspective which focuses on households and the effects of efficiency improvements on consumption patterns. See Freire-González, 2017, Freire-González and Font Vivanco, 2017.

3 For a list of all variable, see Table 7.1 in the Annex. Key terms are explained in Table 7.2.

4 This also follows from the assumption of an input-output framework with given coefficients.

5 This is a standard assumption in post-Keynesian growth theory. See Fazzari and Mott (1986).

6 This proposition assumes that depreciation rates (which are not modelled here) do not differ between various types of capital.

7 There is no inherent reason why capacity utilisation should be the same in all sectors. The point here is merely that the targeted capacity utilisation rates in different sectors are related.

8 In particular in the Kaleckian tradition, it is often assumed that workers consume their income and that only capitalists (i.e. those who receive income from profits) will save. See Lavoie (1992). Since absolute levels of consumption or investment do not matter in the present context, the Kaleckian approach will not be followed.

9 Other values for the mark-up have been investigated but without altering the results substantially.

10 Note that the parameters $a_{ii}$ must be smaller than 1. Otherwise, a capital good would be completely used for its own production.

11 100000 runs are substantially above the number of runs which have been recommended in order to get reliable results. See Mundform et al. (2011).

12 The simulation has been carried out with excel using the YASAI Simulation Add-In, but in principle any other software package such as MATLAB can be used for the same purpose. Note moreover that out of the 100000 runs, approximately 1/3 has led to a positive price vector while in the remaining cases, one or several prices were negative. Since negative prices do not make sense economically, these cases have been ignored.

## Footnotes

13 Results have been inspected visually.
